# Cystic fibrosis in disguise – the wolf in sheep’s clothing, a case report

**DOI:** 10.1186/s12887-021-02636-w

**Published:** 2021-04-14

**Authors:** Friederike Wilbert, Sarah C. Grünert, Andrea Heinzmann, Sebastian F. N. Bode

**Affiliations:** 1 Department of General Pediatrics, Adolescent Medicine and Neonatology, Medical Centre – University of Freiburg, Faculty of Medicine, Freiburg, Germany; 2 Department of Pediatrics and Adolescent Medicine, Ulm University Medical Centre, Ulm, Germany

**Keywords:** Cystic fibrosis, Childhood hypoglycemia, Childhood hepatomegaly, Inborn errors of metabolism

## Abstract

**Background:**

Childhood hypoglycemia in combination with hepatomegaly is suspicious for inborn errors of metabolism. Cystic fibrosis typically presents with failure to thrive, pulmonary and gastrointestinal symptoms. Hepatic involvement and hypoglycemia can occur in a significant number of patients, although hepatomegaly is uncommon.

**Case presentation:**

A 28 months old boy was presented with recurrent upper airways infections, progressive lethargy and weight loss. Clinically hepatomegaly was the main presenting feature and hypoglycemia (minimum 1.4 mmol/l) was noted as were elevated transaminases. The patient did not produce enough sweat to analyze it. Infectious causes for hepatitis were excluded and a broad metabolic work-up initiated. A therapy with starch was initiated to control hypoglycemia. In further course loose stools were reported and pancreatic elastase was found to be reduced. A further sweat test yielded pathological chloride concentration and genetic testing confirmed the diagnosis of cystic fibrosis.

**Conclusions:**

Cystic fibrosis is a systemic disease and less common presentations need to be considered. Even in the age of CF-newborn screening in many countries CF needs to be ruled out in typical and atypical clinical presentations and diagnostics need to be repeated if inconclusive.

## Background

Recurrent hypoglycemia with hepatomegaly is a hallmark of different metabolic disorders, such as glycogen storage diseases, disorders of gluconeogenesis or congenital disorders of glycosylation [1, 2]. However, this combination of symptoms may also occur in cystic fibrosis, a genetic disorder due to mutations in the cystic fibrosis (CF) transmembrane conductance regulator (*CFTR) gene* [3–6]. Most patients with CF present in infancy or childhood with predominantly failure to thrive or pulmonary symptoms such as recurrent pulmonary infections. Nevertheless, manifestations of CF with symptoms beyond the airways and the pancreas are possible and may pose a diagnostic challenge. We herein present the case of a 28 months-old boy with an unusual clinical presentation which was primarily suggestive of an inherited metabolic disorder.

## Case presentation

A 28 months-old boy presented to our emergency department for evaluation of recurrent upper airway infections in the six weeks prior to admission, non-icteric pruritus, and progressive tiredness. He refused to walk although he was described to have been an active child before. He had lost more than 10% of his body weight within 2.5 months. Body weight at presentation was 10.3 kg (5th percentile). Previous history included a heminephrectomy at age 5 months due to a double kidney. The surgery had to be postponed due to a pathologic prothrombin time. As this responded well to a single dose of vitamin K no further evaluation was initiated. Family history was unremarkable.

On clinical examination, hepatomegaly without splenomegaly was noted. The skin was pale; he showed no signs of dehydration and no signs of pathologic coagulation. Clinical examination was normal otherwise. Laboratory parameters at presentation are shown in Table 1. The patient was admitted for further diagnostics and therapy. Infectious causes including CMV, EBV, HSV, hepatitis A-C, and HIV were excluded serologically. Acute phase proteins were normal. No evidence for alpha 1-antitrypsin deficiency, autoimmune hepatitis, or Wilson’s disease was found. Celiac disease was excluded by serological testing. A sweat test yielded no result as the child did not sweat. Vitamin K concentration in serum was again decreased (Table 1), as were other fat-soluble vitamins. Supplementation of vitamin K and other fat-soluble vitamins was started.

**Table 1:** Laboratory parameters at initial presentation. Pathologic parameters are indicated in bold print

Parameter	Result	Normal Range
**Hemoglobin**	**10.5 g/dl**	**10.8–14.3 g/dl**
Leucocytes	16.12 G/μl	5.0–17.0 G/μl
**Prothrombin time**	**38%**	**53–100%**
Partial thromboplastin time	36 s	28–42 s
**Lactate dehydrogenase**	**364 U/l**	**106–296 U/l**
**GOT/AST**	**72 U/l**	**< 20 U/l**
**GPT/ALT**	**69 U/l**	**5–21 U/l**
**Gamma-glutamyl-transferase**	**33 U/l**	**< 20 U/l**
Total bilirubin	0,2 mg/dl	< 1 mg/dl
Alkaline phosphatase	238 U/l	< 281 U/l
**Pancreatic amylasis**	**3 U/l**	**13–60 U/l**
**Lipase**	**6 U/l**	**13–53 U/l**
**Protein**	**5.1 g/dl**	**5.7–8.0 g/dl**
**Albumin**	**2.9 g/dl**	**3.5–5.5 g/dl**
**Lactate**	**2.24 mmol/l**	**0,5–1,6 mmol/L**
Creatine kinase	117 U/l	≤ 370 U/l
Insulin (fasting)	7 pmol/l	18–173 pmol/l (non-fasting)
Blood Glucose	82 mg/dl	74–127 mg/dl
Total cholesterol	55 mg/dl	50–200 mg/dl
**Vitamin K**	**< 0.1** μg**/l**	**0.17–0.68 μg/l**

After admission, severe hypoglycemia (minimum 1.4 mmol/L) was noted. Due to the combination of hepatomegaly, dystrophy and fasting hypoglycemia with only mildly elevated lactate and no cardiac or muscular involvement, a glycogen storage disorder was suspected. A broad metabolic work-up was initiated: Glycogen storage disorders III and IX were excluded. Acid lysosomal lipase activity, a marker for Wolman disease, was within normal ranges. Tyrosinemia type 1 was excluded. Total cholesterol was reduced (Table 1) but a defect of the cholesterol biosynthesis (Smith-Lemli-Opitz syndrome) could be excluded. We performed an isotransferrin electrophoresis to check for congenital disorders of glycosylation. The result was inconclusive, further investigations were initiated and finally, a congenital disorder of glycosylation was ruled out. Hypoglycemia recurred and a diet with frequent carbohydrate-rich meals during the day was initiated. To prevent fasting hypoglycemia, the child was given several doses of uncooked corn starch during the day and at night via nasogastric tube. Hypoglycemia could be controlled and the patient was discharged.

Four weeks later, the patient was admitted with bacterial pneumonia. The parents reported recurrent soft and fatty stools. Elastase was tested in three separate stool samples and was markedly reduced to less than 15 U/L. Sweat test was repeated and showed a markedly increased sweat chloride concentration of 87 mmol/L, which was confirmed by a second sweat test (Cl^−^ 94 mmol/L). Genetic analysis revealed Phe508del (c.1521_1523delCTT) and Arg1066Cys (c.3196C > T) mutations in the *CFTR* gene, confirming the diagnosis of cystic fibrosis.

**Fig. 1:**
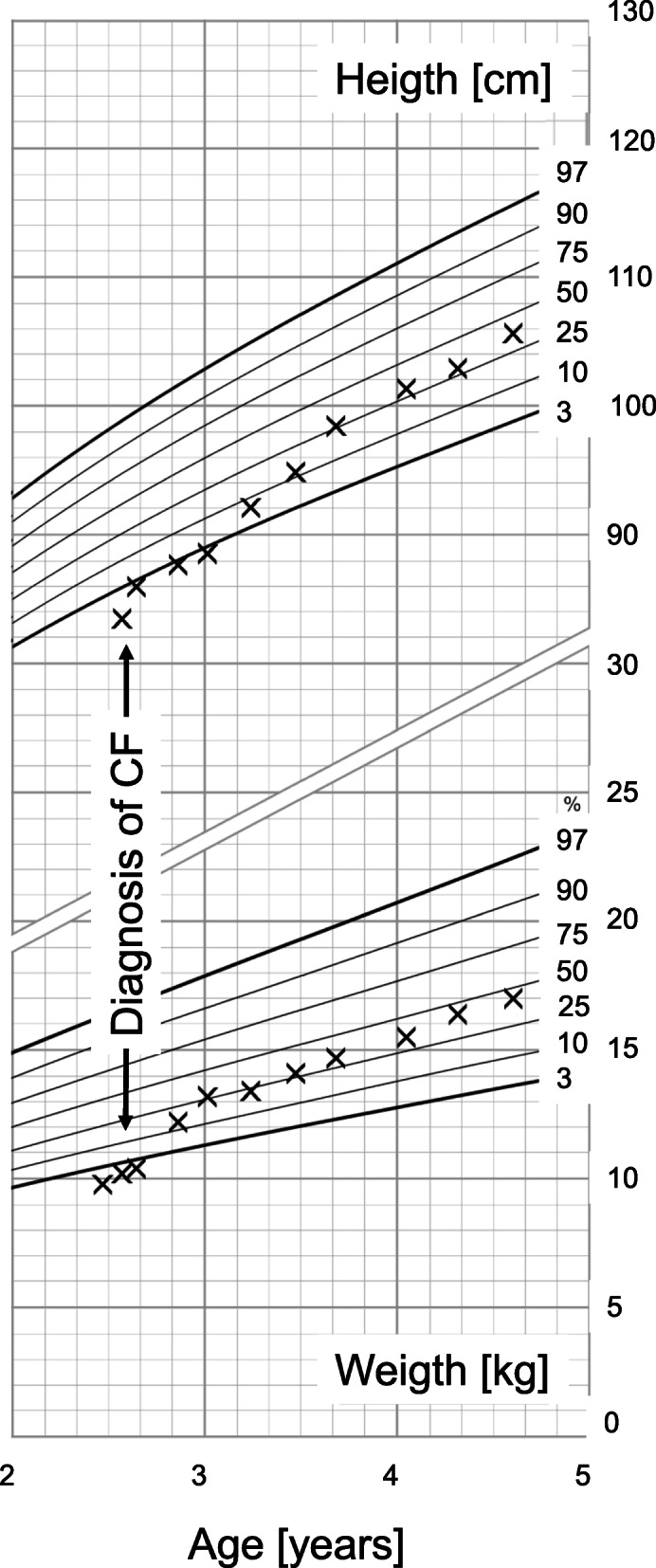
Percentiles (Source: [7]) for height [in cm] and weight [in kg] for the patient. Time of diagnosis of cystic fibrosis is marked

Appropriate therapy with supplementation of pancreas enzymes, inhalation therapy, and physiotherapy was initiated, and vitamin supplementation was continued. Under this regimen appropriate weight gain could be recorded (see Fig. 1). Morning fasting blood glucose normalized and nocturnal corn starch supplementation could be stopped after four months of therapy. Since then, the patient had one pneumonia, treated with oral antibiotics, but otherwise an unremarkable course. Lung function at age 6 years was normal with a Forced Expiratory Volume in 1 s (FEV-1) of 104.4 predicted. Transaminases have been in the normal range for almost five years and hepatomegaly resolved slowly over 9 months.

## Discussion & Conclusion

Cystic fibrosis CF is an autosomal recessive inherited systemic disorder caused by mutations in the CF transmembrane conductance regulator (*CFTR*) gene [3]. CFTR protein is expressed on the apical surface of epithelial cells of the lung, the pancreas, intestines, and biliary ducts leading to the typical clinical picture with pulmonary, but also gastro-intestinal manifestations [3]. Reduced viscosity of bile and pancreatic secretions can cause obstruction and therefore dysfunction of different gastro-intestinal ductal systems causing hepatotoxicity from retained bile components [4]. Hepatic inflammation may initially result in hepatomegaly and later in cirrhosis [4]. CF-associated liver disease has been reported in around 10% of patients with CF [6, 8, 9]. 50% of infants show an intermittent increase in transaminases that spontaneously resolves [10].

Hypoglycemia in CF has been reported, also in patients without CF-induced diabetes and diabetes-directed therapy [11–13]. When measured with a continuous glucose monitoring system, hypoglycemia occurred in more than 3% of the measurement time of three days in 27.5% of patients [13]. In one study with 129 patients with a median age of 17 (range 8–32) years, hypoglycemia was reported in 14% of patients pre-prandially and in 15% postprandially during a standardized oral glucose tolerance test [12, 13]. The underlying causes of hypoglycemia in CF remain unclear but several mechanisms have been postulated including the dysregulation of insulin secretion with inadequate downregulation of insulin secretion in fasting periods [12]. Post-prandial hypoglycemia can occur secondary to delayed and extended insulin response or poor glucagon and incretin responses [13, 14]. Increased energy expenditure, especially during inflammation and infection, may contribute to hypoglycemia, and malnourished patients might especially be at risk [15].

In our patient, most likely the CF-related inflammation plus malabsorption and recurrent upper airway infections in the weeks prior to presentation caused increased inflammation resulting in hepatomegaly, vitamin K deficiency with impaired coagulation, and hypoglycemia. As the combination of hepatomegaly and hypoglycemia is suspicious for inborn errors of metabolism, the diagnostic efforts were focused in this direction, and the correct diagnosis was delayed because the first sweat test yielded no results. On retrospect, all aspects of the case presented here were typical, albeit rare manifestations of cystic fibrosis.

In Conclusion, it is essential to rigorously rule out CF in young children with unclear hepatic involvement and failure to thrive, especially if a first sweat chloride test is inconclusive or does not yield results. While most patients with CF predominantly present with pulmonary symptoms or pancreatic insufficiency, CF needs to be considered a systemic disease with manifestations beyond the airways and the pancreas. Although CF is a target disease of newborn screening programs in many countries, clinical knowledge about different manifesting symptoms of CF is essential and a multi-disciplinary approach is needed to correctly diagnose affected patients. Our patient was born before CF newborn screening was implemented in Germany.

## Data Availability

All data relevant for this case report are included in the manuscript.

## Abbreviations

CF: Cystic fibrosis
CFTR: Cystic fibrosis transmembrane regulator
CMV: Cytomegalovirus
EBV: Epstein-Barr Virus
FEV-1: Forced Expiratory Volume in 1 s
HIV: Human immunodeficiency virus
HSV: Herpes simplex virus
